# Adult Advisor Efficacy to Support LGBTQ+ Youth as a Predictor of LGBTQ+ Youth's Experiences in Gender‐Sexuality Alliances (GSAs) and at School

**DOI:** 10.1002/jad.70111

**Published:** 2026-01-18

**Authors:** V. Paul Poteat, Arthur Lipkin, Cayley C. Bliss, Jonquil Rumberger, Zhengyang Zhong, Robert A. Marx

**Affiliations:** ^1^ Boston College Chestnut Hill Massachusetts USA; ^2^ San Jose State University San Jose California USA

**Keywords:** gender‐sexuality alliances, LGBTQ+ youth, school belonging, social support, victimization

## Abstract

**Introduction:**

Many LGBTQ+ youth seek support from adults at school, which can include advisors of Gender‐Sexuality Alliances (GSAs; clubs affirming LGBTQ+ youth). Little research has considered how efficacious advisors feel to support LGBTQ+ GSA members, or how advisors' efficacy contributes to LGBTQ+ youth's school experiences.

**Methods:**

We examined advisors' self‐efficacy to support and advocate for LGBTQ+ youth (*n* = 30 advisors in 30 schools/GSAs; *M*_age_ = 44.74 years, SD = 10.55; 63% LGBQ+ ; 90% cisgender; 77% White). Utilizing three waves of data from 310 LGBTQ+ youth in these GSAs (*M*_age_ = 15.11 years, SD = 1.60; 98% LGBQ+ ; 55% trans/nonbinary; 50% youth of color), we analyzed three‐level multilevel models to test whether LGBTQ+ youth in GSAs whose advisors reported greater LGBTQ+ self‐efficacy reported more positive experiences within their GSA and in school over 6 months.

**Results:**

Advisors felt competent supporting LGBTQ+ youth, though responses on individual items covered the full range. LGBTQ+ youth in GSAs whose advisors reported greater LGBTQ+ self‐efficacy at the beginning of the study felt that their advisors were more responsive to their needs, perceived a more open and respectful climate in their GSA, and felt greater school belonging over the study period. Advisor LGBTQ+ self‐efficacy did not predict youth′s reported levels of victimization over the study period.

**Conclusions:**

Findings highlight that efficacious GSA advisors could promote safe and inclusive GSAs and schools for LGBTQ+ youth at a time when LGBTQ+ youth face heightened structural oppression, while indicating the need for professional development to enhance advisor efficacy.

## 1 Introduction

LGBTQ+ youth face systemic marginalization in schools, which has negative effects on their health and development (Choukas‐Bradley and Thoma 2022; Russell et al. 2021). Under these conditions, many LGBTQ+ youth seek support from trusted adults at school (Kosciw et al. 2022; Marraccini et al. 2022). They may be especially likely to look to advisors of Gender‐Sexuality Alliances (GSAs), which are school clubs that aim to affirm LGBTQ+ youth (Griffin et al. 2004). Although many GSA advisors serve in this role from a desire to support LGBTQ+ youth (Davis et al. 2024), little research has considered how able they feel to offer guidance and support, or how advisors' efficacy contributes to LGBTQ+ youth's school experiences (Davis et al. 2022). In this study, we examined advisors' sense of competence in their knowledge of LGBTQ+ issues and in their ability to support and advocate for LGBTQ+ youth. Then, over the school year, we examined whether LGBTQ+ youth in GSAs whose advisors reported greater LGBTQ+ self‐efficacy reported more positive experiences in their GSA and school.

### 1.1 The Importance of Adult Support for LGBTQ+ Youth

A majority of LGBTQ+ youth report experiencing and witnessing bias‐based harassment at school (Kosciw et al. 2022), and school policies can also marginalize LGBTQ+ students (e.g., censoring discussion of LGBTQ+ people or issues in course content; ACLU 2023). Youth who experience these minority stressors report lower perceived safety and belonging at school, greater school avoidance and absenteeism, and a range of mental health concerns (Russell et al. 2021). School staff may be important for preventing these experiences or supporting LGBTQ+ youth who face them. For instance, LGBTQ+ youth are more likely to report harassment when they perceive school staff as more supportive (Kosciw et al. 2022). Also, adult mentors can protect LGBTQ+ youth from experiencing forms of minority stress such as bias‐based harassment (Sulimani‐Aidan et al. 2024). As such, GSA advisors could play an outsize role in promoting safer and more welcoming schools for LGBTQ+ youth.

The youth mentorship literature suggests ways in which GSA advisors could support LGBTQ+ youth. Rhodes' (2005) mentorship model proposes that mentors promote youth's wellbeing by providing them with social‐emotional support, scaffolded guidance to facilitate independence, and encouragement to form a deeper understanding of their identity. Central to this model is an adult's ability to form trusting, empathic, and mutual connections with youth. Under these circumstances, adults can help youth process and communicate their feelings with other peers and adults, help youth see their experiences from different perspectives, and offer advice as youth navigate their current circumstances. Mentors can also point to opportunities for youth to develop their interests and provide youth with affirmation of who they are (Rhodes and DuBois 2008). Youth who receive mentoring report better quality relationships with their peers and other adults, greater self‐esteem, lower internalizing concerns, and greater academic engagement (Raposa et al. 2019; Rhodes and DuBois 2008).

Although GSA advisors may not necessarily provide mentorship to LGBTQ+ youth in the same way as youth might receive it in a formal structured mentorship program that pairs youth with adult mentors for one‐on‐one mentoring, they do take on similar roles and provide similar supports in this group setting (Davis et al. 2024), and they could also be comparable to natural mentors (Burningham and Weiler 2021; Edwards et al. 2022). GSA advisors comfort youth who experience rejection; help youth report bullying incidents; act as a liaison between youth and their other teachers or school administrators; connect youth with LGBTQ+ affirming counselors at school or organizations in the local community; identify topics to discuss and co‐facilitate discussions during meetings; and offer scaffolded guidance as youth plan various activities (Davis et al. 2024; Valenti and Campbell 2009; Watson et al. 2010). Despite these myriad roles, GSA advisors have remained largely overlooked in the GSA literature and the broader literature on supportive adults in youth spaces (Sterrett et al. 2011).

Some advisors may feel more competent than others to meet the needs of LGBTQ+ youth in their GSAs. Two earlier studies found that GSA advisors varied in their self‐efficacy to support LGBTQ+ youth (Davis et al. 2022; Poteat and Scheer 2016). Still, we have a limited understanding of how advisors' efficacy could contribute to LGBTQ+ youth's experiences in their GSA or in their school at large. One study found that GSA advisors' efficacy to support transgender students and to discuss transgender issues predicted reduced depressive symptoms among transgender students in GSAs (Andrzejewski et al. 2023). Additional insight could suggest the scope of influence that advisors may have in shaping LGBTQ+ youth's experiences.

### 1.2 Advisor LGBTQ+ Self‐Efficacy and Youth's GSA and School Experiences

We examined several indicators of GSA advisors' LGBTQ+ self‐efficacy. We attended to their reported ability to support LGBTQ+ youth (e.g., in relation to youth's sexual orientation and gender identity), to discuss the unique issues and stressors faced by LGBTQ+ youth (e.g., around experiences of discrimination), and to advocate on behalf of LGBTQ+ youth to others in their school (e.g., to speak up for LGBTQ+ youth to other teachers, administrators, or students). Advisors have named these tasks as central to their role in the GSA (Davis et al. 2024; Valenti and Campbell 2009). Advisors often recognize the need for social‐emotional support among their LGBTQ+ students and for a safe space to solicit this support from peers and trusted adults. Likewise, advisors dedicate a significant amount of their time providing scaffolded guidance as youth plan and conduct awareness‐raising events and engage in advocacy (Poteat et al. 2022). These tasks all align with the roles featured in Rhodes' (2005) mentorship model of providing youth support, scaffolded guidance, and facilitating their identity development.

Advisors' LGBTQ+ self‐efficacy may underlie their ability to provide responsive support to LGBTQ+ youth. Responsiveness reflects one's ability to recognize another person's needs and to respond as that other person would want (Hamre 2014; Reis and Clark 2013). Given the minority stressors that LGBTQ+ youth face (Choukas‐Bradley and Thoma 2022; Goldbach and Gibbs 2017), GSA advisors with a better understanding of these stressors may be more likely to notice them and tailor their support to account for the unique challenges that youth face. This ability may be important for advisors to form trusting and empathic connections with LGBTQ+ youth, which Rhodes' (2005) mentorship model deems essential for effective mentorship.

Advisors may help maintain a respectful climate within GSAs. Youth discuss and act on pressing social issues in their GSAs (Griffin et al. 2004). These conversations and efforts can be challenging and can surface disagreements among members (Calzo et al. 2021; Singh et al. 2024). Advisors with greater LGBTQ+ self‐efficacy may feel better equipped to process or resolve disagreements and ensure that members with different experiences or views feel respected.

Advisors also may help protect LGBTQ+ youth from victimization in the school. Adults are instrumental in setting anti‐bullying norms and enforcing anti‐bullying policies in schools (Ttofi and Farrington 2011). Teachers who report greater self‐efficacy to intervene against homophobic bullying are more likely to do so (Poteat et al. 2019). One recent study found that having a mentor protected LGBTQ+ youth from experiencing various forms of minority stress, including bias‐based harassment (Sulimani‐Aidan et al. 2024). GSA advisors who feel more efficacious in speaking up for LGBTQ+ students and discussing LGBTQ+ issues may have a greater ability to set inclusive norms within the larger school (Valenti and Campbell 2009).

Efficacious advisors may contribute to LGBTQ+ youth's greater sense of belonging at school. School belonging is partly based on a youth's connection to adults in this space (Goodenow 1993), and LGBTQ+ youth may have more meaningful relationships with advisors with greater LGBTQ+ efficacy. Additionally, GSA advisors with greater LGBTQ+ self‐efficacy may feel equipped to advocate for inclusive school policies and practices that affirm LGBTQ+ youth (e.g., using transgender and nonbinary youth's chosen names and pronouns) and they may be able to facilitate youth's own efforts to advocate for such policies and practices (e.g., by providing scaffolded support and guidance as youth engage in advocacy; Davis et al. 2024).

### 1.3 Current Study and Hypotheses

GSA advisors could play a significant role in supporting LGBTQ+ youth and promoting safer and more inclusive schools (Kosciw et al. 2022; Sulimani‐Aidan et al. 2024). In this correlational longitudinal study, we assessed advisors' self‐efficacy to support and advocate for LGBTQ+ youth and explored whether advisors' self‐efficacy predicted LGBTQ+ youth's reported experiences in their GSA and in their school over a 6‐month period with three waves of data. We hypothesized that LGBTQ+ youth in GSAs whose advisors reported greater LGBTQ+ self‐efficacy at Wave 1 would see their advisors as more responsive to their needs and would perceive their GSA as more open and respectful over the study period. We hypothesized that LGBTQ+ youth in GSAs whose advisors reported greater LGBTQ+ self‐efficacy would also report less victimization and feel a greater sense of school belonging over the study period. We adjusted for several covariates, including how long the GSA advisor had been in their role (which could partly reflect their seniority within the school and ability to influence practices that shape youth's school experiences), the size of the GSA (which could contribute to an advisor's ability to provide individualized support), and frequency of GSA meetings (as youth could derive more benefits from GSAs that meet more frequently). We also included whether youth identified as cisgender or trans/nonbinary, as research indicates that trans/nonbinary youth report greater experiences of minority stress than cisgender youth (Day et al. 2018).

## 2 Methods

### 2.1 Participants

Participants in the current study included 310 LGBTQ+ youth (*M*_age_ = 15.11 years, SD = 1.60) who were members of their school's GSA and 30 adult advisors of these GSAs (*n* = 30 schools/GSAs; *M*_age_ = 44.74 years, SD = 10.55). The GSAs were in high schools (*n* = 25), middle schools (*n* = 3), or combined middle and high schools (*n *= 2). With the exception of one youth participant who identified as heterosexual (and who identified as transgender), all other youth participants (cisgender and trans/nonbinary participants) reported LGBQ+ identities. They participated as part of a larger project on the experiences of youth in GSAs and were based in Massachusetts, Northern and Southern California, and New York City. Table 1 provides demographic information for the youth and advisor participants.

**Table 1:** Youth and Adult Advisor Demographics and Descriptive Statistics for Measures.

Variable	*N*	*M* (SD)	Range
**Student demographics and measures**			
Sexual orientation^a^			
Gay or lesbian	54		
Bisexual or pansexual+	100		
Questioning	21		
Heterosexual	1		
Asexual	32		
Queer	24		
2 or more identities (excluding heterosexual)	70		
2 or more identities (including heterosexual)	3		
Not reported	5		
Gender Identity^b^			
Cisgender male	25		
Cisgender female	74		
Questioning+	12		
Non‐binary+	35		
Gender queer+	2		
Gender fluid+	25		
Agender+	7		
Bigender+	9		
Masculine gender expansive+	21		
Feminine gender expansive+	19		
Gender expansive+	19		
Transgender (no other gender identities specified)	2		
Transgender male	18		
Transgender female	3		
Not codable	1		
Not reported	38		
Race or ethnicity			
White or European American	139		
Black or African American	14		
Asian or Asian American	32		
Latina/o/x	51		
Middle Eastern, Arab, or Arab American	2		
Biracial or multiracial	56		
Not reported	16		
Age		15.11 (1.60)	11.00–19.00
Open respectful climate (Wave 1)		4.38 (0.67)	1.00–5.00
Open respectful climate (Wave 2)		4.42 (0.56)	3.00–5.00
Open respectful climate (Wave 3)		4.39 (0.67)	2.00–5.00
Victimization (Wave 1)		1.74 (2.31)	0.00–12.00
Victimization (Wave 2)		1.03 (1.63)	0.00–7.00
Victimization (Wave 3)		1.16 (1.76)	0.00–8.00
School belonging (Wave 1)		3.31 (0.93)	1.00–5.00
School belonging (Wave 2)		3.29 (1.01)	1.00–5.00
School belonging (Wave 3)		3.17 (0.96)	1.00–5.00
Advisor responsiveness (Wave 1)		4.36 (0.70)	1.29–5.00
Advisor responsiveness (Wave 2)		4.37 (0.69)	2.43–5.00
Advisor responsiveness (Wave 3)		4.29 (0.73)	2.00–5.00
**Advisor demographics and measures**			
Sexual orientation			
Gay or lesbian	7		
Bisexual or pansexual+	7		
Questioning	1		
Heterosexual	11		
2 or more identities (excluding heterosexual)	4		
Gender identity			
Cisgender male	9		
Cisgender female	18		
Non‐binary+	1		
Feminine gender expansive	2		
Race or ethnicity			
White or European American	23		
Black or African American	2		
Asian or Asian American	1		
Latina/o/x	3		
Not reported	1		
Age		44.74 (10.55)	26.00–64.00
Number of years as GSA advisor		5.56 (2.59)	0.25–10.50
Number of GSA members		17.97 (7.00)	5.00–35.00
Frequency of GSA meetings			
Every other week	7		
Once per week	22		
More than once per week	1		
Advisor LGBTQ+ self‐efficacy (Wave 1)		3.76 (0.58)	1.57–4.50

*Note:* Student and advisor demographic information was self‐reported. Descriptive data for the following measures were student‐reported: advisor responsiveness, open and respectful GSA climate, victimization, and school belonging. Descriptive data for the following measures were advisor‐reported: LGBTQ+ self‐efficacy, estimated number of GSA members, and frequency of GSA meetings.

^a^ When identifying their sexual orientation identities, youth had the option to “mark all that apply” from the response options, including a write‐in option. “Bisexual or Pansexual +” represents participants who described their sexual orientation identity by selecting “Bisexual” and/or “Pansexual,” and/or who wrote in responses that indicated sexual or romantic attractions to more than one sex and gender (e.g., omnisexual, panromantic, polysexual).

^b^ Youth responded to two questions to identify their gender identities. The first question provided participants the option to “mark all that apply” from current gender identity options, including a write‐in option, and the second question asked whether participants identified as transgender (at Wave 2) or their sex assigned at birth (Wave 1). When possible, we used youth's responses to the gender and transgender identity items to place them into these categories, but when we did not have information from youth on the transgender identity item, we used their reported sex assigned at birth to place them into the categories. Because of the large number of response patterns, we classified youth into a smaller number of more broadly inclusive groups for the purpose of their presentation in Table 1. “Questioning +” includes participants who marked “Questioning” for their gender identity and/or in response to the question of whether they identified as transgender, regardless of the number of additional identities selected. “Non‐binary +,” “Gender queer +,” “Gender fluid +,” and “Agender +” describe participants who marked only those identities for their gender identity, and any response option to the second gender identity question. “Bigender +” describes participants who selected “male” and “female,” or who wrote in “bigender,” regardless of the number of additional identities selected and their response to the second gender identity item. “Masculine gender expansive + ” describes participants who identified as male and one or more additional gender identities (including write‐in responses), who identified as male and transgender and one or more other gender identities, or who provided a write‐in response that signaled a form of transmasculine gender expression (e.g., “boy flux”). “Feminine gender expansive +” followed the same categorization logic as “masculine gender expansive +.” “Gender expansive +” describes youth who did not select male or female, but either selected “yes” to the transgender item and more than one the non‐binary gender identity responses (e.g., gender queer) or who wrote in “pangender”.

The current participant sample was drawn from the original sample of 627 youth and 70 advisors of 51 GSAs who had participated in the project. The sample was reduced to include only youth with LGBTQ+ identities (*n* = 553; 88% of the original sample), given the current study's focus on advisors' LGBTQ+ self‐efficacy in relation to LGBTQ+ youth's GSA and school experiences. The sample was also restricted to GSAs with only one advisor (59% of the total GSAs, resulting in the final sample of 310 youth in the current study [49% of the original sample]), which we did for two reasons. First, youth were asked about their advisor's responsiveness without the ability to distinguish among multiple advisors, which would make it difficult to interpret the meaning of the scores or the findings for GSAs with multiple advisors. Second, there was no clear way to treat multiple efficacy scores from advisors within a single GSA to predict youth's experiences: averaging them or using the highest score would either produce an incomplete, skewed, or potentially uninterpretable mixed portrayal of efficacy and mask how advisors may take on different roles or interact differently with youth in the GSA based on their efficacy. Advisors of GSAs not included in the current study did not differ from advisors of GSAs included in the current study on their LGBTQ+ self‐efficacy (*p* = 0.95) or on how long they had been an advisor (*p* = 0.96). A multivariate analysis indicated that LGBTQ+ youth in GSAs not included in the current study did not differ from LGBTQ+ youth in GSAs included in the current study on the three waves of their reports of their advisors' responsiveness, the open and respectful climate of their GSAs, their experiences of victimization, or their sense of school belonging (*p* = 0.22).

Among the current sample of participating LGBTQ+ youth, 51 (21%) participated at all three waves, 51 (21%) participated at two waves, and 141 (58%) participated at one wave. LGBTQ+ youth who participated in more waves reported greater perceptions of an open and respectful climate in their GSA at Wave 1 (*r* = 0.18, *p* = 0.005), but youth's number of waves of participation were not correlated with their reports of advisor responsiveness, victimization, or school belonging (*p*s = 0.10, 0.55, and 0.65, respectively). Participation rates also did not differ between cisgender and trans/nonbinary youth (*p* = 0.47) or between white youth and youth of color (*p* = 0.22).

### 2.2 Procedures

We collected data during the 2021 and 2022 school years, with efforts to include GSAs in a diverse range of schools located in rural, suburban, and urban areas, and that varied in racial diversity and size. GSA advisors provided adult consent in place of parent consent in Massachusetts and Northern California. This is an increasingly recommended practice in LGBTQ+ youth research to minimize risk of outing youth to unsupportive caregivers (Macapagal et al. 2017). Parent consent was required in New York City and Southern California. In these locations, the consent form described the study as having a general interest in student club involvement, without specifically naming Gender‐Sexuality Alliances. In all locations, youth provided their assent to participate after receiving adult consent, and GSA advisors also gave their consent prior to completing their own survey. Youth completed their three surveys over a roughly 6‐month period of the school year. After youth in a GSA completed their first survey, the second wave of data collection was planned to occur following a two‐ to 3‐month period. The same spacing was followed for the third wave of data collection. The ranging window of time between waves was due to our accommodations for school breaks and testing periods, scheduling visits that were convenient for the GSA, or from providing a several‐week window for completing the online version of the survey. Youth and advisors were offered $10 for each completed survey. All procedures were approved by the institutional review board of Boston College (protocol 19.280.01).

### 2.3 Measures

#### 2.3.1 Youth Demographics

We report LGBTQ+ youth's demographic information in Table 1, including their sexual orientation, gender identity, race or ethnicity, and age. We asked youth to “Check all you would use to describe your sexual orientation,” and provided the following options: gay or lesbian, bisexual, questioning, heterosexual/straight, pansexual, asexual, queer, or feel free to be more specific (with write‐in space provided). There were many response patterns, and so we engaged in an intensive coding process using youth's responses to present the sexual orientation identities of participants parsimoniously in Table 1 for descriptive purposes. We sought to create a smaller number of categories while still preserving relative accuracy and alignment with youth's responses.

To assess youth's gender identity, we used a two‐step process (National Academies of Sciences, Engineering, and Medicine 2022). At Wave 1, we asked youth to “Check all you would use to describe your gender identity,” with response options including: male, female, questioning, nonbinary, genderqueer, gender fluid, agender, or feel free to be more specific (with write‐in space provided). We also asked youth to “Check the sex you were assigned at birth,” with response options including: male, female, intersex/other. At Wave 2, based on emerging recommendations for respectfully asking youth about transgender identity, we asked youth whether they identified as transgender rather than their sex assigned at birth (“Some people describe themselves as transgender when the way they think or feel about their gender is different from their sex assigned at birth. Do you identify as transgender?”) with response options including: no, yes, or I'm not sure yet or I'm questioning. We still included the first item on gender identity. We engaged in an intensive coding process to parsimoniously present youth's gender identities in Table 1 for descriptive purposes.

Finally, we asked youth to “Check all you would use to describe your racial/ethnic identity,” and provided the following options: Black or African American, Asian/Asian American, Latino/a/x, Bi/Multiracial, Native American, Middle Eastern/Arab or Arab‐American, or feel free to be more specific (with write‐in space provided). We coded the many response patterns to arrive at the groups we present for descriptive purposes in Table 1.

#### 2.3.2 Advisor Demographics

We report GSA advisor demographic information in Table 1, including their sexual orientation, gender identity, race or ethnicity, and age. We also asked advisors to report how long they had served as a GSA advisor, how often their GSA met, and the number of students they estimated to be regular active members of the GSA (see Table 1).

#### 2.3.3 Advisor LGBTQ+ Self‐Efficacy

At Wave 1, advisors responded to 14 items that we generated as a research team that asked about their efficacy to support LGBTQ+ youth, discuss the unique issues and stressors faced by LGBTQ+ youth, and advocate on behalf of LGBTQ+ youth. Items were preceded by the stem, “How competent do you currently feel to do the following?” Sample items included: Support transgender and nonbinary students on issues related to gender identity and expression; advocate or speak up on behalf of transgender and nonbinary students to other teachers or administrators at the school; talk about unique experiences that LGBQ students face; and, address issues and topics related to sexual orientation. Response options were *not at all*, *a little*, *moderately*, *quite a bit*, and *extremely* (scored 1 to 5). All items are presented in Table 2. Higher average scale scores represent greater LGBTQ+ self‐efficacy (*α* = 0.91).

**Table 2:** Descriptive Statistics for Advisor LGBTQ+ Self‐Efficacy Items.

How competent do you currently feel to do the following?	*M* (SD)	Reported range
1. Talk about unique experiences that transgender and non‐binary students face	2.70 (0.95)	1–4
2. Address issues and topics related to gender identity and expression	3.20 (1.00)	1–5
3. Explain the difference between gender identity and sexual orientation	3.80 (0.81)	2–5
4. Support transgender and non‐binary students on issues related to gender identity and expression	3.27 (0.83)	2–5
5. Take actions to address instances of bullying or discrimination that transgender and non‐binary students face	3.40 (0.86)	1–5
6. Advocate or speak up on behalf of transgender and non‐binary students to other students at the school	3.67 (0.80)	1–5
7. Advocate or speak up on behalf of transgender and non‐binary students to other teachers or administrators at the school	3.63 (0.85)	1–5
8. Talk about unique experiences that LGBQ students face	3.97 (0.85)	2–5
9. Address issues and topics related to sexual orientation	4.11 (0.83)	2–5
10. Take actions to address instances of bullying or discrimination that LGBQ students face	4.10 (0.92)	1–5
11. Talk about students' experiences from different sexual orientation identities	3.93 (0.79)	2–5
12. Support LGBQ students on issues related to sexual orientation	4.17 (0.79)	2–5
13. Advocate or speak up on behalf of LGBQ students to other students at the school	4.40 (0.81)	1–5
14. Advocate or speak up on behalf of LGBQ students to other teachers or administrators at the school	4.33 (0.92)	1–5

*Note:* Reported range represents the actual range of scores reported by advisors for each item; the possible range for all items was 1–5.

#### 2.3.4 Advisor Responsiveness

At all three waves, youth completed the 7‐item Care subscale from the Tripod study (Ferguson and Danielson 2014) to indicate their advisor's level of responsiveness to their needs so far that year at Wave 1 and over the past 2 months at Waves 2 and 3 (e.g., “I liked the way my GSA advisor treated me when I needed help”). Response options range from *totally untrue* to *totally true* (scored 1 through 5). Higher average scale scores indicate greater perceived advisor responsiveness (*α*_Level1_ = 0.81; *α*_Level2_ = 0.95; *α*_Level3_ = 0.94).

#### 2.3.5 Open and Respectful Climate of the GSA

At all three waves, youth completed the 4‐item Open Classroom Climate scale (Flanagan et al. 2007) to indicate the extent to which they perceived their GSA to have an open and respectful climate so far that year at Wave 1 and over the past 2 months at Waves 2 and 3. Items were preceded by the stem, “In our GSA, students…” with items including: (a) have a voice in what happens; (b) can disagree with the advisor if they are respectful; (c) can disagree with each other if they are respectful; and (d) are encouraged to express opinions. Response options range from *strongly disagree* to *strongly agree* (scored 1 to 5). Higher average scale scores represent perceptions of a more open and respectful climate in the GSA (*α*_Level1_ = 0.79; *α*_Level2_ = 0.94; *α*_Level3_ = 0.94).

#### 2.3.6 Victimization

At all three waves, youth completed three items about their experiences of peer victimization in the past 30 days. Items were preceded by the stem, “How often did these things happen to you in the last 30 days, in school or out of school,” and items included: I got hit or pushed by other students; other students picked on me, made fun of me, or called me names; and, other students left me out or excluded me. Response options were *0 times*, *1 or 2 times*, *3 or 4 times*, *5 or 6 times*, or *7 or more times* (scored 0 through 4). Higher total scale scores indicate experiencing greater peer victimization (*α*_Level1_ = 0.45; *α*_Level2_ = 0.80; *α*_Level3_ = 0.85).

#### 2.3.7 School Belonging

At all three waves, youth completed six items from the Psychological Sense of School Membership scale (Goodenow 1993) to indicate their sense of school belonging so far that year at Wave 1 and currently at Waves 2 and 3 (e.g., “I feel like I matter to people at my school,” and “Other students here like the way I am”). Response options range from *totally untrue* to *totally true* (scored 1 through 5). Higher average scale scores represent a greater sense of school belonging (*α*_Level1_ = 0.81; *α*_Level2_ = 0.96; *α*_Level3_ = 0.95).

### 2.4 Analytic Approach

We tested four identical multilevel models, one for each outcome of interest (advisor responsiveness, open and respectful GSA climate, victimization, and school belonging), using multilevel modeling in Mplus 8.3 (Muthén and Muthén 2017). Our models thus accounted for the interdependence of observations (Level 1) nested within individuals (Level 2) who were nested within GSAs (Level 3). We used maximum likelihood with robust standard error estimation and full information maximum likelihood (FIML) to handle the missing data so that all available data are used in the analyses, including from participants with missing data (as opposed to excluding these participants entirely). The results from FIML produce comparable or near identical results to multiple imputation (Lee and Shi 2021), which is not available for 3‐level models.

At Level 1 (the within‐individual level) of our models, we included a variable for the wave of data collection in order to adjust for potential variability in our dependent variables tied to any successive linear change over time during the study period. We scaled the variable such that Wave 1 was treated as the intercept (i.e., scored as 0), and Waves 2 and 3 were scored as 1 and 2, respectively. At Level 2 (the between‐individual level), we included youth's gender, coded as cisgender or trans/nonbinary (with cisgender youth as the reference group, and essentially comparing cisgender LGBQ+ and trans/nonbinary LGBQ+ youth with exception of one transgender youth who identified as heterosexual), to account for variability between youth based on their gender identity in the dependent variables over the study period. At Level 3 (the between‐GSA/school level), we included advisors' LGBTQ+ self‐efficacy to account for differences across GSAs in members' reports on the dependent variables over the study period. We also included the additional covariates of how long advisors had served in their advising role (grand‐mean centered), the advisor's sexual orientation, gender identity, and race/ethnicity (each dichotomized such that 0 = heterosexual and 1 = LGBQ+ for sexual orientation; 0 = cisgender and 1 = trans/nonbinary for gender identity; 0 = White and 1 = racial or ethnic minority identities for race/ethnicity), how often their GSA met (0 = once every other week, 1 = once per week or more), and the number of students they estimated to be regular active members of the GSA (grand‐mean centered).

Finally, we conducted sensitivity analyses in which we analyzed our models with the data that also included LGBTQ+ youth in GSAs with more than one advisor. To do so, we had to modify the coding of several variables, including advisor sexual orientation, gender identity, race/ethnicity, how long advisors had served in their advising role, and the advisor LGBTQ+ self‐efficacy score due to the fact that the responses from multiple advisors in the same GSA were not always identical. In these models, we coded these variables to indicate whether at least one advisor identified as LGBQ+ (for sexual orientation), whether at least one advisor identified as trans/nonbinary (for gender identity), whether at least one advisor identified as a person of color (for race/ethnicity), the average length of advising among advisors, and the average LGBTQ+ self‐efficacy among advisors (for these variables, the values used in the analyses for GSAs with one advisor remained unchanged). In these sensitivity analyses, advisor LGBTQ+ self‐efficacy was only significant in the model for school belonging, and these results are reported in Supplemental Table S1. Because of the additional coding complexities of the covariates alongside the ambiguity of coding the advisor LGBTQ+ efficacy variable that we mentioned previously, we focus on the analyses utilizing the more restricted sample of GSAs with one advisor. At best, the sensitivity analyses indicate that our results should not yet be considered generalizable beyond GSAs with one advisor.

## 3 Results

### 3.1 Preliminary Descriptive Analyses

Based on their average LGBTQ+ self‐efficacy scores, advisors generally felt competent in providing support, discussing unique stressors, and advocating on behalf of LGBTQ+ youth. Responses on individual items, however, nearly covered the full possible range. Still, the average scores for individual items fell within the range of moderately competent to extremely competent, based on the response option anchors. Descriptive information on advisors' responses to these self‐efficacy items can be found in Table 2. Univariate analyses of variance (ANOVAs) indicated no statistically significant differences between LGBQ+ advisors and heterosexual advisors, *F* (1, 28) = 0.002, *p* = 0.97, or between white advisors and advisors of color, *F* (1, 27) = 0.86, *p* = 0.36, on their LGBTQ+ self‐efficacy.

We calculated intraclass correlation coefficients (ICCs) to determine the proportion of total variance in youth‐reported advisor responsiveness, open and respectful GSA climate, victimization, and school belonging at the within‐individual, between‐individual, and between‐GSA/school levels. For advisor responsiveness, 7% of the total variance was at Level 3 (i.e., between GSAs/schools), while 53% was at Level 2 (i.e., between individuals) and 40% was at Level 1 (i.e., within individuals). For GSA climate, 7% of the total variance was at Level 3, 41% was at Level 2, and 52% was at Level 1. For victimization, 9% of the total variance was at Level 3, 50% was at Level 2, and 41% was at Level 1. For school belonging, 12% of the total variance was at Level 3, 52% was at Level 2, and 36% was at Level 1.

### 3.2 Primary Analyses of Multilevel Models

The results of our multilevel models are reported in Table 3 (for GSA‐based experiences) and Table 4 (for broader school‐based experiences). In our model for advisor responsiveness, as hypothesized, LGBTQ+ youth in GSAs whose advisors reported greater LGBTQ+ self‐efficacy at Wave 1 perceived their advisor to be more responsive to their needs over the 6‐month study period (*γ* = 0.70, *p* < 0.001). There were also statistically significant covariate effects. There was a slight decrease in perceived advisor responsiveness over the study period (π = −0.11, *p* = 0.05), trans/nonbinary youth reported lower advisor responsiveness than cisgender youth (*β* = −0.27, *p* < 0.001), and youth in smaller GSAs and in GSAs that met more frequently reported greater advisor responsiveness (GSA size: *γ* = −0.42, *p* = 0.02; GSA meeting frequency: *γ* = 0.46, *p* = 0.01).

**Table 3:** Multilevel Model Results for Youth's GSA Experiences.

Independent variables	Advisor responsiveness		Open respectful climate	
	Coefficient	95% CI	Coefficient	95% CI
Level 1 (within individuals)				
Time	−0.11*	(−0.21, −0.002)	−0.05	(−0.15, 0.06)
Level 2 (between individuals)				
Gender identity	−0.27***	(−0.42, −0.13)	−0.18**	(−0.29, −0.02)
Level 3 (between GSAs/schools)				
Advisor LGBTQ+ self‐efficacy	0.70***	(0.43, 1.00)	0.47*	(0.07, 1.00)
Advisor tenure	−0.12	(−0.39, 0.16)	−0.11	(−0.49, 0.38)
GSA number of members	−0.42*	(−0.76, −0.08)	0.05	(−0.35, 0.58)
GSA meeting frequency	0.46**	(0.14, 0.77)	0.64***	(0.38, 1.00)
Advisor sexual orientation	0.22	(−0.21, 0.65)	−0.10	(−0.53, 0.45)
Advisor gender	−0.02	(−0.38, 0.34)	−0.28	(−0.69, 0.25)
Advisor race/ethnicity	−0.17	(−0.64, 0.30)	−0.45	(−1.04, 0.32)

*Note:* Values represent standardized coefficient estimates and their 95% confidence intervals (95% CI). Gender identity was coded as 0 = cisgender youth and 1 = trans/nonbinary youth. Advisor sexual orientation, gender, and race/ethnicity were binary indicators, where 0 = heterosexual, cisgender, and white for the respective variable, and 1 = LGBQ+ , trans/nonbinary, and person of color for the respective variable. Advisor tenure = length of time served as GSA advisor in years.

*** *p* < 0.001

** *p* < 0.01

* *p* < 0.05.

**Table 4:** Multilevel Model Results for Youth's Broader School‐Based Experiences.

Independent variables	Victimization		School belonging	
	Coefficient	95% CI	Coefficient	95% CI
Level 1 (within individuals)				
Time	−0.17**	(−0.28, −0.06)	−0.13**	(−0.23, −0.04)
Level 2 (between individuals)				
Gender identity	0.10	(−0.04, 0.24)	−0.31***	(−0.44, −0.17)
Level 3 (between GSAs/schools)				
Advisor LGBTQ+ self‐efficacy	−0.001	(−0.51, 0.51)	0.50*	(0.07, 0.94)
Advisor tenure	−0.18	(−0.90, 0.53)	−0.23	(−0.77, 0.31)
GSA number of members	−0.23	(−0.59, 0.13)	0.03	(−0.42, 0.50)
GSA meeting frequency	−0.10	(−0.54, 0.33)	0.40	(−0.56, 0.41)
Advisor sexual orientation	−0.03	(−0.55, 0.50)	−0.02	(−0.55, 0.51)
Advisor gender	−0.41*	(−0.75, −0.08)	0.16	(−0.12, 0.44)
Advisor race/ethnicity	−0.44**	(−0.73, −0.14)	−0.19	(−0.55, 0.16)

*Note:* Values represent standardized coefficient estimates and their 95% confidence intervals (95% CI). Gender identity was coded as 0 = cisgender youth and 1 = trans/nonbinary youth. Advisor sexual orientation, gender, and race/ethnicity were binary indicators, where 0 = heterosexual, cisgender, and white for the respective variable, and 1 = LGBQ+ , trans/nonbinary, and person of color for the respective variable. Advisor tenure = length of time served as GSA advisor in years.

*** *p* < 0.001

** *p* < 0.01

* *p* < 0.05.

In our model for the open and respectful climate of the GSA, as hypothesized, LGBTQ+ youth in GSAs whose advisors reported greater LGBTQ+ self‐efficacy at Wave 1 perceived their GSA to have a more open and respectful climate over the study period (*γ* = 0.47, *p* = 0.02). Several covariate effects were statistically significant. Trans/nonbinary youth reported lower perceptions of an open and respectful climate in the GSA than cisgender youth (*β* = −0.18, *p* = 0.003), and youth in GSAs that met more frequently reported greater perceptions of an open and respectful climate in the GSA (*γ* = 0.64, *p* < 0.001).

In our model for victimization, there was not a significant association between advisor LGBTQ+ self‐efficacy and LGBTQ+ youth's experiences of victimization over the study period (*γ* = −0.001, *p* = 0.99). There were several statistically significant covariate effects. Youth reported a decrease in victimization over the study period (π = −0.17, *p* = 0.003), and youth in GSAs whose advisors identified as trans/nonbinary (*γ* = −0.41, *p* = 0.01) and youth in GSAs whose advisors identified with racial or ethnic minority identities (*γ* = −0.44, *p* = 0.004) reported lower victimization.

In our model for school belonging, there was a significant association between advisor LGBTQ+ self‐efficacy and LGBTQ+ youth's sense of school belonging. LGBTQ+ youth in GSAs whose advisors reported greater LGBTQ+ self‐efficacy at Wave 1 felt a greater sense of school belonging over the study period (*γ* = 0.50, *p* = 0.02). Two covariate effects were statistically significant. Youth reported a decrease in school belonging over the study period (π = −0.13, *p* = 0.006), and trans/nonbinary youth reported a lower sense of school belonging than cisgender youth (*β* = −0.31, *p* < 0.001).

## 4 Discussion

Our findings suggest the potential benefits, but also the likely bounds, of GSA advisors' contributions to LGBTQ+ youth's experiences in their GSA and in their school through their efficacy to support LGBTQ+ youth. Advisors' greater LGBTQ+ self‐efficacy predicted more positive experiences for LGBTQ+ youth within the GSA, but the effects were less consistent for youth's experiences in the school at large. These findings point to the need for a greater focus on how GSA advisors can provide the most impactful support for LGBTQ+ youth both within GSAs and in the broader school context.

### 4.1 Advisor Variability in LGBTQ+ Self‐Efficacy

There was some variability among GSA advisors in their reported LGBTQ+ efficacy. The average reported self‐efficacy score fell between the competency markers of “moderate” to “quite a bit,” suggesting that advisors did, on average, feel competent in their abilities to discuss LGBTQ+ issues and to support and advocate for LGBTQ+ youth. Indeed, all average scores for individual items, except for one, fell within the markers of “moderate” to “extremely” competent. Of note, this variability was not tied to advisors' sexual orientation or race/ethnicity. These descriptive findings are understandable in some ways, as advisors volunteer for this position (Valenti and Campbell 2009) and may select such a position partly out of a sense of competence. Additionally, some GSA advisors are approached to serve in the role by youth who likely seek adults to serve as advisors whom they view as more competent than others. Still, it could be informative for future research to consider how advisors with different lived experiences and constellations of privileged and marginalized identities develop a sense of efficacy in similar and unique ways. In the general population of school staff, it may be more likely that differences in LGBTQ+ self‐efficacy exist on the basis of adults' sexual orientation or gender identity. Yet, as we go on to note, even among a sample of advisors who generally felt efficacious about their LGBTQ+ knowledge and skills, their variability in efficacy did relate to variability in LGBTQ+ youth′s reported GSA and school experiences.

Variability in advisors' LGBTQ+ efficacy could be tied to a range of other factors. Advisors with a background in health‐related fields (e.g., counseling, nursing, or social work) may have had more academic preparation in supporting youth who face marginalization (Abreu et al. 2022). Advisors also may have sought additional opportunities to build their competence through workshops or continuing education courses (Davis et al. 2022). Future research should consider how these and other factors may build advisors' competence and indirectly improve LGBTQ+ youth's experiences in their GSA and at school.

### 4.2 Advisor LGBTQ+ Efficacy and Youth's GSA‐Related Experiences

Advisors' LGBTQ+ self‐efficacy predicted LGBTQ+ youth's proximal experiences within their GSA. This was reflected in youth's perceptions of their advisor's responsiveness to their needs and their perceptions of the GSA's climate. We had hypothesized these associations based on Rhodes' (2005) youth mentorship model, which highlights the important role of adults in providing youth with support, scaffolded guidance, and encouragement to explore their identity. Our findings suggest that advisors can contribute to several kinds of experiences that youth have in GSAs, tied in part to their knowledge and skills specifically around supporting LGBTQ+ youth.

Youth in GSAs whose advisors reported greater LGBTQ+ self‐efficacy at the beginning of the study felt that their advisors were more responsive to their needs over the 6‐month study period. This finding adds nuance to extant research showing that advisors take on their role from a desire to support LGBTQ+ youth (Davis et al. 2024; Valenti and Campbell 2009). Our finding suggests that while this may be the case, it does not mean that all advisors are equally prepared to respond effectively to the needs that LGBTQ+ youth bring to GSAs. Some youth bring needs for social‐emotional support following parental rejection or peer harassment, others need referrals to supportive resources at school or in the community, and others seek opportunities to engage in advocacy (Davis et al. 2022; Poteat and Scheer 2016). Advisors who felt more knowledgeable about and able to discuss LGBTQ+ stressors and needs may have been better able to notice and ask LGBTQ+ youth about these experiences (e.g., rejection from a family member; McCurdy et al. 2023) and to offer support. Furthermore, these advisors may have been better able to respond effectively to the broad range of needs that youth brought to their GSA. Youth also may have felt more comfortable disclosing their concerns to these advisors. These abilities may have enabled efficacious advisors to establish trusting and empathic connections with youth in their GSA, which Rhodes' (2005) mentoring model posits to be essential for effective mentoring. Ultimately, LGBTQ+ youth may have felt that these advisors provided them the specific kind of support that they were seeking.

Advisors' greater LGBTQ+ self‐efficacy also predicted youth's perceptions of a more open and respectful climate in their GSA over the study period. Although GSAs aim to provide a safe setting for youth to socialize and seek support (Griffin et al. 2004; Poteat et al. 2017), youth in GSAs come with different experiences of privilege and marginalization, and often discuss issues that can activate strongly held feelings and beliefs (Calzo et al. 2021; Singh et al. 2024). These issues can include youth's experiences of interlocking forms of oppression (e.g., discrimination against LGBTQ+ youth who are also undocumented; Singh et al. 2024) or issues of injustice within different LGBTQ+ communities (e.g., for LGBTQ+ youth of color; Calzo et al. 2021). Although many GSAs are youth‐led, youth have suggested that advisors should take initiative to facilitate these discussions (Singh et al. 2024), and advisors may have been in a unique position to notice larger group dynamics or tensions emerge among members. Advisors with greater LGBTQ+ self‐efficacy may have been better equipped to process disagreements or ensure that youth with different views felt respected (e.g., when discussing different experiences of individuals within the LGBTQ+ community; Poteat et al. 2019; Singh et al. 2024). This aligns with key provisions of mentoring outlined within Rhodes' (2005) mentorship model, namely the ability of adults to offer guidance and aid to youth as they learn to communicate their feelings to others and to understand others' different perspectives.

### 4.3 Advisor LGBTQ+ Efficacy and Youth's School‐Related Experiences

The associations between GSA advisors' LGBTQ+ self‐efficacy and LGBTQ+ youth's broader experiences within the school were less consistent. We considered broader school experiences in relation to youth's perceptions of school belonging and their experiences of victimization. Our findings here provide a sense of the potential limits to a GSA advisor's sphere of influence in promoting the safety of and respect toward LGBTQ+ youth within the larger school context.

There was an association between GSA advisors' LGBTQ+ self‐efficacy and LGBTQ+ youth's perceptions of school belonging over the 6‐month study period. From an ecological lens, we had expected that LGBTQ+ youth's sense of school belonging would be tied to their relationships with their advisor—which could be more positive with more efficacious advisors—given that GSA advisors represent the school through their formal positions in it. We had also expected that advisors with greater LGBTQ+ self‐efficacy would be better able to advocate on behalf of LGBTQ+ youth (or support LGBTQ+ youth's advocacy) for inclusive policies and practices that could promote a welcoming school climate. In this respect, advisors with greater LGBTQ+ self‐efficacy may have been better able to do this work in the face of hostile responses from other adults at the school (Valenti and Campbell 2009). Still, the scope of influence of one GSA advisor on the views and actions of other school personnel and students may be limited, especially in the current sociopolitical context of open hostility toward efforts aimed at cultivating equity and inclusion and with anti‐LGBTQ+ legislation aimed at schools (ACLU 2023). Future research should consider how GSA advisors work in partnership with other adults or students within or outside the GSA to cultivate a more welcoming climate wherein LGBTQ+ youth feel they belong.

Finally, GSA advisors' LGBTQ+ self‐efficacy did not relate to LGBTQ+ youth's reported levels of victimization over the study period. This runs counter to some research suggesting that mentors can protect against victimization and minority stressors among LGBTQ+ youth (Sulimani‐Aidan et al. 2024). One‐on‐one mentoring may be more effective at this than the mentoring that GSA advisors can provide in a group setting. This role of preventing victimization is not one that is as explicitly covered in Rhodes' (2005) mentoring model, which focuses more on providing support or cultivating hope for youth as they experience challenges. When considered alongside the significant association between advisors' efficacy and youth's perceptions of advisors' responsiveness to their needs, advisors with greater LGBTQ+ self‐efficacy may have been more able to respond to victimization but no more able to prevent it. Bullying prevention programs often involve ecologically‐minded school‐wide implementation (Bradshaw 2015), and even then, their effects can be small (Ng et al. 2022). Thus, it may be unrealistic to expect that a single GSA advisor, even one with high self‐efficacy, would have the capacity to fully protect LGBTQ+ youth from victimization. Whereas prior research has tended to focus on adults' efficacy to intervene against bullying or their motivation to support LGBTQ+ youth experiencing victimization (Fischer et al. 2021; Valenti and Campbell 2009), our findings underscore the need for more research on effective strategies that GSA advisors can learn and implement to prevent victimization from occurring.

### 4.4 Additional Individual and GSA Covariate Associations of Importance

Although this study focused on GSA advisors' LGBTQ+ self‐efficacy and its ties to LGBTQ+ youth's experiences, we briefly note several individual and GSA covariate associations of importance. Trans/nonbinary youth (of whom all except for one individual identified as LGBQ+ in our study) tended to report less positive experiences within their GSA and in their school at large than their cisgender LGBQ+ peers. This could be due to several factors. GSA advisors vary in their self‐efficacy to meet the needs of trans/nonbinary youth (Andrzejewski et al. 2023; Poteat and Scheer 2016). Moreover, both during and since the time of our data collection in 2021 and 2022, many anti‐LGBTQ+ policies directed toward schools have expressly targeted trans/nonbinary students (McQuillan et al. 2024). Our findings align with those from the minority stress literature that have documented the negative effects of structural oppression (e.g., living in states with discriminatory laws and policies) on the health and wellbeing of LGBTQ+ youth (English et al. 2022; Fields and Wotipka 2022) and that trans/nonbinary youth report greater levels of minority stress than cisgender youth at school (Day et al. 2018; Kosciw et al. 2022). Although we were unable to make finer comparisons for trans/nonbinary youth who identified as heterosexual relative to trans/nonbinary youth who identified as LGBQ+, our findings still emphasize the need to identify how adults, allied peers, and other school‐based resources can best support trans/nonbinary youth at school.

There were also several significant covariate associations tied to GSA size and meeting frequency. GSA meeting frequency was associated with youth's reports of greater advisor responsiveness and perceptions of a more open and respectful GSA climate, which warrant further consideration. Although some GSAs meet weekly, others meet less frequently or consistently (Davis et al. 2024). This may pose challenges for establishing supportive norms within the GSA or for advisors to respond to members' concerns. These findings could also result from reciprocal processes (e.g., GSAs also may meet less frequently following disagreements or less respectful interactions among members). We also found that youth in smaller‐sized GSAs felt their advisor was more responsive to their needs. Given the multidimensional aims of GSAs and varied needs that youth bring (Adelman et al. 2022; Griffin et al. 2004), it could be increasingly challenging for advisors to be responsive to a larger number of youth, regardless of their LGBTQ+ efficacy.

### 4.5 Strengths, Limitations, and Future Directions

Our study is characterized by several strengths in its contribution to the literature. We gave attention to the adult advisors of GSAs, who are understudied but key contributors to these groups. Furthermore, we linked data from advisors to youth's data on their experiences both within the GSA and in the broader school setting to consider the proximal and more distal ways in which GSA advisors could provide support, protection, and foster more affirming spaces for LGBTQ+ youth. Finally, whereas research on GSA advisors and youth members has mostly relied on cross‐sectional data, we utilized multiple waves of data from youth about their GSA and school experiences to consider how advisors' LGBTQ+ efficacy could relate to youth's experiences over a 6‐month period. This allowed us to account for fluctuations in youth's own experiences in their GSA and at school and to consider more prolonged associations between advisors' LGBTQ+ efficacy and these experiences.

We also highlight some limitations that could qualify our findings. We included GSAs with one advisor, but not multiple advisors, due to the measurement and analytic complications noted in our methods. Our sensitivity analyses that included GSAs with multiple advisors only yielded significant results for school belonging, which could be due to the inherent coding decisions and limitations that came with analyzing these data. Given that many GSAs have multiple advisors (about 40% in our study), this is an important limitation for future research to address and to ensure that measures are inclusive and applicable for GSAs with multiple advisors. Such research should discern whether our current findings generalize to these GSAs, while also addressing unique questions relevant to these GSAs. Additionally, we were unable to consider how GSA advisors' LGBTQ+ self‐efficacy related to the experiences of LGBTQ+ youth who were not in the GSA. Also, although we constructed a measure of advisors' LGBTQ+ self‐efficacy with items that aligned with advisor roles in GSAs and roles featured in Rhodes' (2005) mentorship model, our sample of 30 advisors kept us from conducting any rigorous evaluation of the measure. It would be valuable for future research to focus on developing measures for adult advisors as part of a larger effort to bring more adequate attention to, and assessment of, their contributions in GSAs and their needs in this role. Another measurement limitation: although our primary focus was on advisor efficacy associations at the between‐GSA level, the reliability estimate for victimization at the within‐individual level was low. Future research aiming to identify predictors of within‐individual variability in LGBTQ+ youth's victimization may wish to utilize a measure with stronger psychometric properties at this level of analysis. Furthermore, there was significant attrition in our youth sample, despite our use of multiple strategies to retain them (e.g., offering remuneration for each survey, providing the opportunity to complete the survey online within a several‐week window for each wave, maintaining regular communication with advisors and sending reminders to them and youth about each survey). This could limit the generalizability of our findings, and future research should aim to replicate and extend our work with larger and more diverse samples of LGBTQ+ youth. Finally, we considered associations between advisors' LGBTQ+ efficacy at the beginning of the study and LGBTQ+ youth's experiences over a 6‐month period, but future research should also consider how changes in advisors' efficacy over time may coincide with changes in youth's experiences.

In all, our findings highlight the need for greater attention to adult advisors in GSAs and how their skills and contributions to GSAs could promote positive experiences for LGBTQ+ youth. The current sociopolitical moment in which anti‐LGBTQ+ policies have been proposed and enacted that target schools (ACLU 2023) underscores the necessity of having GSAs and adults in schools who feel equipped and able to support and advocate for LGBTQ+ youth. As many anti‐LGBTQ+ campaigns and policies are targeting the school setting, there is even more need to support GSA advisors and the other adults in schools who seek to ensure school safety and affirmation for LGBTQ+ youth.

## Author Contributions

**V. Paul Poteat:** writing – original draft, conceptualization, formal analyses, funding acquisition, investigation, methodology. **Arthur Lipkin:** writing – review and editing, conceptualization. **Cayley C. Bliss:** writing – review and editing, data curation. **Jonquil Rumberger:** writing – review and editing. **Zhengyang Zhong:** writing – review and editing. **Robert A. Marx:** writing – review and editing, conceptualization, investigation, methodology.

## Ethics Statement

All procedures were in accordance with the ethical standards of the institutional research committee and with the 1964 Helsinki declaration and its later amendments or comparable ethical standards. All study procedures were approved by the Boston College IRB, protocol 19.280.01.

## Conflicts of Interest

The authors declare no conflicts of interest.

## Supporting information


**Supplemental Table S1:** *Sensitivity Analysis Results for Multilevel Models.*

## Data Availability

Due to the current sociopolitical climate and direct targeting and marginalization of LGBTQ+ young people by individuals and institutions, including misrepresentation of data and research on the health and wellbeing of LGBTQ+ young people, the data from the present study are not publicly accessible. However, the analytic code, research materials, and further descriptive analyses of the data are available upon request.
