# Supplementary material for: Adult Advisor Efficacy to Support LGBTQ+ Youth as a Predictor of LGBTQ+ Youth's Experiences in Gender‐Sexuality Alliances (GSAs) and at School
Source: J Adolesc. 2026 Jan 18;98(3):965–77. doi: 10.1002/jad.70111 (PMC13044863; doi:10.1002/jad.70111)
Supplement: Supplementary file 1 — Supplemental Table S1: Sensitivity Analysis Results for Multilevel Models. [file JAD-98-965-s001.docx]

**Supplemental Table S1**

*Sensitivity Analysis Results for Multilevel Models*

|  | Advisor Responsiveness | |  | Open Respectful Climate | |  | Victimization | |  | School Belonging | |
| --- | --- | --- | --- | --- | --- | --- | --- | --- | --- | --- | --- |
| Independent Variables | Coefficient | 95% CI |  | Coefficient | 95% CI |  | Coefficient | 95% CI |  | Coefficient | 95% CI |
| Level 1 (within individuals) |  |  |  |  |  |  |  |  |  |  |  |
| Time | -0.12** | (-0.20, -0.03) |  | -0.06 | (-0.14, 0.02) |  | -0.07 | (-0.18, 0.04) |  | -0.14*** | (-0.22, -0.07) |
| Level 2 (between individuals) |  |  |  |  |  |  |  |  |  |  |  |
| Gender identity | -0.25*** | (-0.36, -0.13) |  | -0.13* | (-0.24, -0.03) |  | 0.20** | (0.08, 0.32) |  | -0.27*** | (-0.37, -0.17) |
| Level 3 (between GSAs) |  |  |  |  |  |  |  |  |  |  |  |
| Avg. adv. LGBTQ+ efficacy | 0.53 | (-1.14, 2.21) |  | 0.42 | (-0.04, 0.88) |  | -0.07 | (-0.48, 0.34) |  | 0.46** | (0.13, 0.78) |
| Avg. adv. tenure | 0.16 | (-0.61, 0.92) |  | 0.16 | (-0.08, 0.40) |  | -0.14 | (-0.56, 0.28) |  | -0.08 | (-0.37, 0.21) |
| GSA number of members | -0.48* | (-0.92, -0.04) |  | -0.31 | (-0.72, 0.10) |  | -0.03 | (-0.44, 0.39) |  | -0.11 | (-0.42, 0.21) |
| GSA meeting frequency | 0.50 | (-1.01, 2.00) |  | 0.69*** | (0.36, 1.01) |  | 0.03 | (-0.42, 0.49) |  | -0.14 | (-0.56, 0.29) |
| At least 1 LGBQ+ adv. | 0.28 | (-0.54, 1.10) |  | -0.08 | (-0.49, 0.33) |  | -0.29 | (-0.89, 0.30) |  | 0.04 | (-0.25, 0.53) |
| At least 1 TNB adv. | -0.04 | (-0.55, 0.48 ) |  | -0.11 | (-0.51, 0.28) |  | -0.47* | (-0.85, -0.09) |  | 0.14 | (-0.13, 0.41) |
| At least 1 adv. of color | -0.35 | (-1.11, 0.41) |  | -0.32 | (-0.81, 0.16) |  | -0.14 | (-0.57, 0.29) |  | -0.19 | (-0.53, 0.14) |
| Number of adv. | 0.05 | (-0.27, 0.37) |  | 0.17 | (-0.17, 0.50) |  | 0.26 | (-0.18, 0.71) |  | -0.16 | (-0.50, 0.18) |

*Note*. Values represent standardized coefficient estimates and their 95% confidence intervals (95% CI). Avg. = average, Adv. = advisor; TNB = trans/nonbinary. Gender identity was coded as 0 = cisgender youth and 1 = trans/nonbinary youth. Advisor sexual orientation, gender, and race/ethnicity were binary indicators, where 0 = heterosexual, cisgender, and white for the respective variable, and 1 = LGBQ+, trans/nonbinary, and person of color for the respective variable. Advisor tenure = length of time served as GSA advisor in years.

*** *p* < .001; ** *p* < .01; * *p* < .05
